# Effect of random and hub gene disruptions on environmental and mutational robustness in *Escherichia coli*

**DOI:** 10.1186/1471-2164-7-237

**Published:** 2006-09-18

**Authors:** Tim F Cooper, Andrew P Morby, Annabel Gunn, Dominique Schneider

**Affiliations:** 1 School of Biological Sciences, University of Auckland, Auckland, New Zealand; 2 School of Biosciences, Cardiff University, Cardiff, UK; 3 Laboratoire Adaptation et Pathogénie des Microorganismes, Université Joseph Fourier, Institut Jean Roget, F-38041 Grenoble, France

## Abstract

**Background:**

Genome-wide profiling has allowed the regulatory interaction networks of many organisms to be visualised and the pattern of connections between genes to be studied. These networks are non-random, following a power-law distribution with a small number of well-connected 'hubs' and many genes with only one or a few connections. Theoretical work predicts that power-law networks display several unique properties. One of the most biologically interesting of these is an intrinsic robustness to disturbance such that removal of a random gene will have little effect on network function. Conversely, targeted removal of a hub gene is expected to have a large effect.

**Results:**

We compared the response of *Escherichia coli *to environmental and mutational stress following disruption of random or hub genes. We found that disruption of random genes had less effect on robustness to environmental stress than did the targeted disruption of hub genes. In contrast, random disruption strains were slightly less robust to the effect of mutational stress than were hub disruption strains. When we compared the effect of each disruption on environmental and mutational stress, we found a negative relationship, such that strains that were more environmentally robust tended to be less robust to mutational stress.

**Conclusion:**

Our results demonstrate that mutant strains of *E. coli *respond differently to stress, depending on whether random or hub genes are disrupted. This difference indicates that the power-law distribution of regulatory interactions has biological significance, making random disruptions less deleterious to organisms facing environmental stress. That *E. coli *can reduce the effect of environmental stress without reducing the phenotypic effect of additional mutations, indicates that robustness and evolvability need not be antagonistic.

## Background

Genome-wide profiling has enabled researchers to organize the regulatory interactions of many organisms into global networks detailing connections between genes. To date most research has focused on describing the overall pattern formed by these connections, typically finding them to follow a power-law distribution comprising a small number of well-connected 'hub' genes and many genes with one or a few connections [reviewed in 1]. Now, as an increasing number of these networks have been described, the implications of their organization is being considered. For example, does network topology place evolutionary constraints on component nodes [2-4] and what general biological consequences emerge from power-law regulatory distributions [4-8]?

Theoretical work predicts that power-law networks possess an intrinsic robustness to perturbation because removal of a randomly chosen node will have little effect on network topology and thus little impact on network function [9,10]. The rationale behind this prediction is that power-law networks are characterized by a relative paucity of highly connected hub nodes, therefore a random deletion is unlikely to effect one of these hubs. In contrast, targeted deletion of a highly connected hub node is expected to have a significant effect on the ability of the network to function. However, these predictions are based on analysis of computational networks, and it is not clear whether they will apply to biological networks [6,11].

There are at least two reasons to be cautious before extending the structure-function relationship predicted in computational networks to biological networks. First, it is not always clear how to assess the effect of disrupting a node on the function of a network. Computational studies have considered topological measurements (e.g. the average number of connections required to bridge random pairs of nodes) or the ability to reach regulatory equilibrium, as a measure of function [9,10,12-14]. However, while this capacity is clearly an important functional attribute of most biological organisms, many other attributes also contribute to organism fitness. Only if all these attributes correlate with one another can any one be used as a proxy for network function. Second, unlike computational networks, nodes in biological networks have an intrinsic function; even genes with few connections can be essential for cell survival if they encode a necessary biochemical reaction.

Several recent studies address the first of the points discussed above by taking advantage of a collection of all viable single gene deletion yeast strains to measure the effect of each deletion on growth rate [4,5,7,8]. Relating the growth rate of each deletion strain to the topological attributes (e.g. the number of connections made with other genes) of the deleted gene allows a direct examination of the effect of these attributes on organism function. These studies have shown that highly connected genes are more likely to be essential [4,7] and have a tendency toward having a more deleterious effect on growth rate when deleted [5,8]. However, this latter relationship is relatively weak, and may result from biases in the network interaction data sets [11]. A possible reason for this weak relationship is outlined in the second point mentioned above. That is, deletion of a gene imposes both a general network topology, and a unique biochemical, defect on the mutant strain. Without distinguishing between these effects, it is not possible to isolate the role of network topology in determining organism function.

One way to, at least partially, disentangle the topological and biochemical effects of gene deletions, is to consider the relative effect of deletions with and without an additional stress. In practice, this involves comparing the effect of a gene disruption on an organism's growth rate (or some similar measure of fitness) in a reference environment and in the same environment to which some additional stress – either external (i.e. environmental) or internal (i.e. mutational) – is added. Because most aspects of the environment remain unchanged, any constant biochemical effect of the deletion is 'cancelled out', leaving topological change as the cause of growth rate differences in response to stress. For example, deletion of a regulatory gene will have pleiotropic effects, removing a number of links in an organism's regulatory network. As long as the main biochemical demands of the reference and stressful environment are similar, differences in growth rate will result mostly from changes in the ability of the deletion strain to regulate genes in response to the stress, rather from the loss of biochemical activities of the deleted genes. Of course, it is not possible to rule out the possibility that some genes will have different biochemical effects in response to different environmental stresses. However, a recent study, which carried out a normalization step similar to that outlined above, found that only a very small fraction of genes (<~5%) had different effects on growth of the yeast *Saccharomyces cerevisiae *in more than two of 21 environments screened [15]. This result suggests that the biochemical utility of most genes remains reasonably constant across a range of environments.

In this study, we tested whether the power-law organization of the *Escherichia coli *transcriptional network confers an intrinsic robustness to disruption [16]. To do this, we created a series of isogenic strains differing only in the disruption of either randomly chosen or hub genes. To estimate the effect of these disruptions on robustness we measured the growth rate of disruption strains, relative to the reference strain, in response to various stresses, both external (environmental) and internal (mutational). Robustness was calculated as the reduced sensitivity of a strain to a particular stress. Following Montville et al (2005)[17], this was done in two ways: as the mean effect of stress on each disruption strain and as the variation in the response of each strain over all stresses. A higher (i.e. more detrimental) mean effect or a higher variance are expected to be related, and both would indicate reduced robustness [18,19]. We present results of both variance and mean derived robustness because both measures are important in considering possible selection for power-law interaction networks. Comparing these results across random and hub disruption strains allowed us to address whether the power-law organization of the transcriptional network confers robustness to random disruptions.

## Results

### Effect of random and hub disruptions

**Figure 1:**
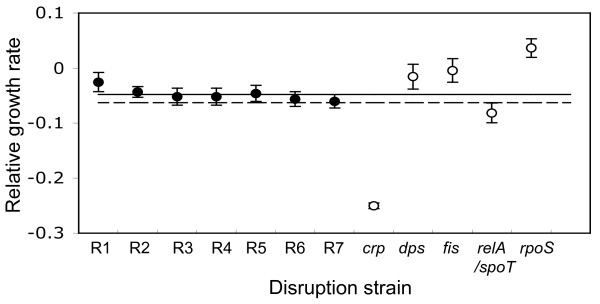
Effect of random and hub mutations on growth in the reference environment. The effect of each disruption was measured as the log ratio of the growth rate of the disruption strain to that of the reference strain. Random disruption strains, solid symbols; Targeted hub disruption strains, hollow symbols. Error bars indicate 95% confidence intervals. Solid line indicates mean of random disruption strains; dotted line indicates mean of targeted hub disruption strains.

The mean effect of random and hub gene disruptions was determined by measuring the growth rate of each disruption strain within these two groups (figure 1). Growth rates were measured in a rich medium, LB, which served as a reference environment throughout this experiment. Disruption of random genes tended to be less deleterious than the disruption of hub genes (figure 1). However, a nested analysis of variance showed that this difference was not significant (*F*_1,10 _= 0.179, *P *= 0.681). Because growth rate is only measured in one environment, it was not possible to estimate variation in the response of any disruption strain.

One explanation for the lack of a mean effect of disruption type on growth rate is the high variation between the different hub disruption strains. Additional analyses of variance were performed separately on random and hub disruption strains to estimate the variation between strains within each group. These analyses found that variation in growth rate was substantially higher between the different hub disruption strains than between random disruption strains (supplementary tables 1 and 2 [see Additional file 1]). Variance components of the strain effect were 0.014 and 4.9 × 10^-5 ^in the hub and random disruption type analyses, respectively. Taking the square root of these values allows us to calculate that the average growth rate difference between hub disruption strains was ~11.7% compared to ~0.7% between random disruption strains. This high variation between hub disruption strains illustrates the importance of factoring out the specific biochemical effects of disruptions before their general topological effect can be examined.

### Environmental robustness

**Figure 2:**
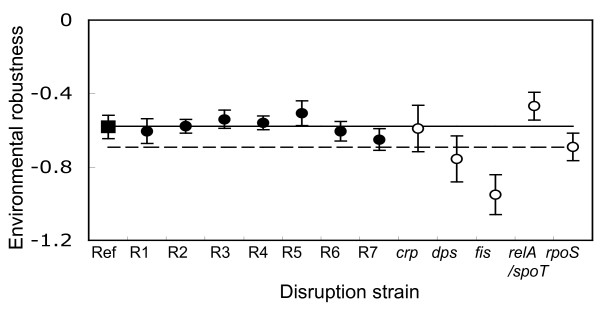
Robustness of random and hub deletion mutants to environmental stress. Average of median values measured in each environment is plotted for each strain. Error bars indicate standard errors. Reference strain (ref), solid square, random disruption strains, solid circles; targeted hub disruption strains, hollow circles. Solid line indicates mean of random disruption strains; dotted line indicates mean of targeted hub disruption strains.

To detect differences in the robustness of disruption strains to environmental stress we measured the growth rate of each strain in each of fifteen stressful environments (Materials and Methods) (figure 2). Each measurement was paired with a measurement of the same strain in the reference environment. Taking the log ratio of these measurements allowed us to normalize measurements, factoring out the specific biochemical effect of a mutation from its general effect on response to stress.

We measured robustness in response to stress in two ways: the amount of variation in the response to multiple stresses and the mean effect of these stresses. An effect of disruption type (hub or random) on variation in growth rate can be tested by analyzing the significance of the variation among strains response to environmental stress nested within disruption type. To do this, we analyzed growth rate using mixed linear models that were able to fit different variances for random factors. We found significantly higher variance in growth rate amongst strains with hub gene disruptions (table 2, supplementary table 3 [see Additional file 1]). To estimate this difference directly, we performed separate analyses of variance for the hub and random disruption strains (supplementary tables 4 and 5 [see Additional file 1]). The resulting estimates of the within disruption type variance components were 0.154 and 0.041 for hub and random disruption strains, respectively. These values correspond to an average difference in growth rate between stress environments of ~39% amongst hub disruption strains, compared to ~20% amongst random disruption strains. We note that there was not a significant relationship between the effect of the original gene disruption and the average response to stress (R^2 ^= 0.114, F_1,10 _= 1.291, *P *= 0.282). Therefore, the difference in response of hub and random strains cannot be explained as a consequence of any systematic tendency of strains with higher or lower growth rates to respond differently to environmental stress.

Considering the mean response of genotypes, the main effect of disruption type was not significant, however, its interaction with environment was highly significant (table 2 and supplementary table 3 [see Additional file 1]). Therefore, although there was no overall effect of disruption type on mean response to stress, this response did vary over environments in a way that depended on disruption type.

### Mutational robustness

**Figure 3:**
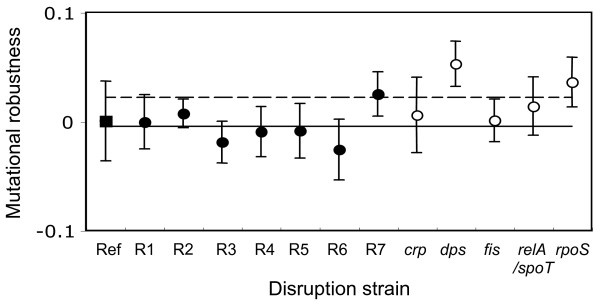
Robustness of random and targeted hub deletion mutants to mutational stress. Average of median values for each of the 30 secondary mutations made in each strain is plotted. Error bars indicate standard errors. Reference strain (ref), solid square; random disruption strains, solid circles; targeted hub disruption strains, hollow circles. Solid line indicates mean of random disruption strains; dotted line indicates mean of targeted hub disruption strains.

To test the effect of disruption type on response to mutational stress, we constructed a series of thirty secondary mutation-containing strains from each disruption strain. Each of these derivative strains differed from the original strain by the presence of a single transposon insertion, giving a total of 390 (13 (reference + 12 disruption) strains × 30 secondary insertions) secondary mutation-containing strains. As above, we measured the growth rate of each of these strains and used a mixed model to ask whether the primary hub or random disruption had any impact on the effect of the additional mutations (figure 3). We did not find any evidence for differences in the amount of variation in the effect of secondary mutations amongst hub and random disruption strains (table 2). However, there was a marginally significant effect of disruption type on the mean effect of secondary mutations (table 2 and supplementary table 6 [see Additional file 1]). The mean effect of secondary mutations corresponded to a relative change in growth rate of 0.022 ± 0.009 (± SE) in the hub disruption strains and of -0.004 ± 0.007 (± SE) in the random disruption strains. Thus, as a group, random disruption strains appear to be slightly less robust than hub disruption strains. There was not a significant relationship between the effect of the initial disruption and subsequent mutations on growth rate (R^2 ^= 0.045, F_1,10 _= 0.473, *P *= 0.507). Therefore, this result was not due to any systematic tendency for secondary mutations to have an effect depending on the growth rate of the original disruption strain.

### Relationship between environmental and mutational robustness

**Figure 4:**
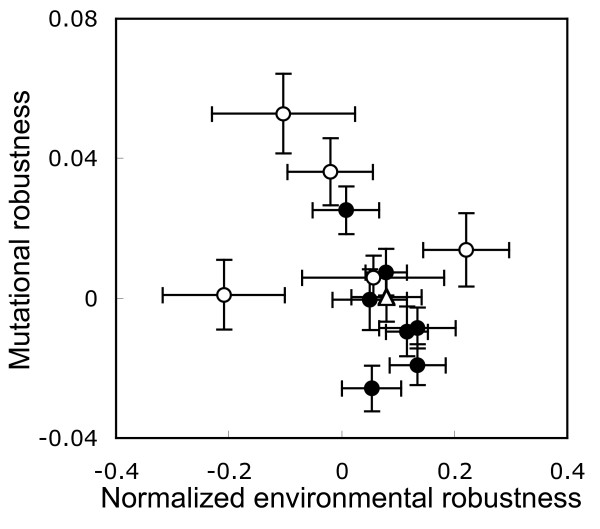
Relationship between environmental and mutational robustness. Because different environmental stresses had different mean effects on growth rate we normalized the effect of each stress to have a mean of zero. Therefore values on this axis above zero do not indicate that the effect of stress was positive. Average of median values plotted. Error bars indicate standard errors. Reference strain, hollow triangle; random disruption strains, solid circles; targeted hub disruption strains, hollow circles.

To assess the similarity in response to environmental and mutational stress, we compared the average effect of each stress type for each disruption strain (figure 4). If similar mechanisms underlie the effect of each stress type, we expected a positive correlation between environmental and mutational robustness. In fact, the correlation between the effect of the perturbations on growth rate was negative, though this relationship was not significant (Pearson, r = -0.413, *P *= 0.161). Visual inspection of the data indicated that this relationship may have been biased by an outlying point corresponding to the *fis*-deleted strain, which had much lower environmental robustness than any other disruption strain. When we repeated the analysis using a non-parametric test (Kendall, tau b = -0.359, *P *= 0.088), or omitting this strain (Pearson, r = -0.644, *P *= 0.032), the negative relationship between environmental and mutational robustness became much stronger. Together, these results indicate a tendency for more environmentally robust strains to be less mutationally robust.

## Discussion

We tested the expectation that power-law networks are robust to random disruption of component nodes by comparing the effect of environmental and mutational stresses on hub and random gene disruptions in *E. coli *. Because random disruptions are unlikely to cause large topological changes in power-law distributions, we expected these disruptions to have little effect on network function, and thus robustness, relative to the effect of deleting highly-connected hub genes. Two metrics were used to assess robustness, the mean effect of stress, and the variation in this effect across independent stresses. Our main findings can be summarised as follows. 1. There was no consistent effect of disruption type on growth rate in a reference environment. 2. The effect of environmental stress was less variable among random disruption strains, indicating they were more robust. 3. The mean effect of secondary mutations was greater amongst random disruption strains, indicating they were less mutationally robust. 4. There was a negative relationship between environmental and mutational robustness such that disruption strains that were more robust to environmental stress tended to be less robust to mutational stress. Below we discuss each of these findings in turn.

### No consistent effect of disruption type on growth rate in a reference environment

A comparison of average growth rate in the reference environment found no difference in the effect of hub and random gene disruptions. Hub disruption genes were chosen on the basis of the high number of interactions they made in the transcriptional network of *E. coli*. Therefore, in line with theoretical expectations, we expected that they would tend to be more deleterious [9,10]. A possible reason we did not observe this is that nodes in biological networks, unlike in most computational networks, have unique functions that may mask a general effect of their deletion on network function. Our results for the *rpoS *disruption strain illustrate this complication. Despite affecting a large number of regulatory interactions, this strain had a growth advantage relative to the ancestor in our reference environment (*t *= 2.744, df = 84, *P *= 0.007). RpoS alters the specificity of RNA polymerase, directing transcription for a suite of genes involved in starvation response [20]. Several previous studies have shown that *rpoS *null mutants have a growth advantage under various environmental conditions, possibly because of increased activity of a competing factor, RpoD, that may coordinate expression of a more suitable suite of genes [21-24]. Thus, any general fitness effect of the *rpoS *deletion mediated through changes in network topology are likely to be masked by the direct biochemical effect of its deletion.

### Random disruption strains were more robust to environmental stress

To avoid the potentially confounding effects of simultaneously measuring the biochemical and network effects of gene disruptions, we focused on measuring the ability of disruption strains to respond to stress. By measuring this response relative to the effect of the disruption in a reference environment, we hoped to reduce the biochemical effect of the disruption, which should be similar across environments [15]. Considering the mean response of hub and random disruption strains over 15 environmental stresses, we found that the random disruption strains had higher growth rates than the hub disruption strains, but that this difference was not significant. A possible explanation for this lack of significance is the variation in the mean response of the different disruption types to different environmental stresses revealed by the significant interaction between environment and disruption type. One way to reduce the influence of this variation, is to ask whether there was a general tendency for random disruption strains to have higher growth rates. Random disruption strains had a higher mean growth rate in 11 of the 15 environments tested. This difference is higher than expected by chance, indicating that the effect of environmental stress tended to be less deleterious in random disruption strains (one-tailed binomial test, *P *= 0.030). This conclusion complements our finding that variation in response over all stresses was significantly lower in the random disruption strains. Together these results indicate that random disruption strains were significantly more robust to environmental stress then were hub disruption strains.

### Random disruption strains were less robust to mutational stress

Disruption type did not explain any of the variation in growth rate amongst the 30 secondary mutation strains constructed from each primary disruption strain. However there was a marginally significant mean effect. Surprisingly, this difference corresponded to a positive mean effect of secondary mutations on hub disruption strains compared to a small negative effect on random disruption strains. To look at this result in more detail we analyzed the individual and mean effect of the secondary insertions separately for each disruption strain (data not shown). We found that only the *dps*-deleted strain had a significant mean increase in growth rate measured over all insertion derivative strains (mean effect of secondary insertion mutations = 0.057, two-tailed *t*-test, *H *= 0, *t *= 5.540, df = 28, *P *< 0.001). Although individual insertion mutations significantly increased growth rate in several other primary disruption strains, in no case were these increases significant following a sequential Bonferroni correction, performed to account for the multiple comparisons made.

The finding of an increase in average growth rate resulting from secondary insertion mutations in the *dps*-deleted strain was unexpected because it is usually assumed that most mutations, especially large mutations such as transposon insertions, will be deleterious. Indeed, a previous study that measured the average effect of 226 insertion mutations in a strain derived from the one used here, found an average fitness cost of 2.75% [26]. One potentially important difference between the study mentioned above, and the one reported here, is the use of a different assay environment. The study by Elena et al (1998), measured fitness in a minimal glucose environment, where a relatively large fraction of genes are probably involved in metabolism and growth. In contrast, we measured the effect of insertion mutations in LB medium, a rich environment that provides the majority of precursors needed for *E. coli *to grow. Consequently, a larger fraction of genes are likely to be functionally redundant in this environment and the average effect of insertion mutations is likely to be smaller.

A possible explanation for the mean beneficial effect of secondary mutations in the *dps*-deleted strain is the occurrence of compensatory mutations during the construction of the secondary mutation-containing strains. By reducing the deleterious effect of either the *dps *deletion or of a secondary insertion mutation, such mutations would increase the growth rate of the secondary mutation-containing strains, even if the effect of the secondary mutation was itself deleterious [27]. Although we took care in the construction of the secondary mutation strains to reduce selection for compensatory mutations (see Materials and Methods), we cannot rule out that they may have occurred. To assess the possibility that compensatory mutations influenced our results, we performed several additional analyses of the growth rate measurements of the hub disruption strains. First, we asked if there was a correlation between the effect of the initial gene deletion and the mean fitness effect of the secondary mutations. The compensatory mutation scenario predicts a negative correlation, because deletions that cause the biggest initial decrease in fitness provide the strongest selection for compensatory mutations that recover this fitness loss. In fact, the relationship between original deletion effect and average effect of secondary mutations was positive and non-significant (r = 0.486, *P *= 0.407).

Second, we examined the distribution of effects on growth rate caused by secondary mutations in the *dps*-deleted strain. A null expectation of this distribution is hard to determine, therefore we used the Kolmogorov-Smirnov test to directly compare this distribution between the *dps*-deleted and reference strains. This comparison is instructive because, in the absence of a primary disruption mutation, compensatory mutations are not available to the reference strain. Differences between these two distributions therefore provide a sensitive test to reveal the possible influence of compensatory mutations. We found that the distribution of secondary mutation effects in the *dps*-deleted strain was significantly different from that of the reference strain (D = 0.633, *P *< 0.001). Furthermore, this difference was more extreme than in comparisons of any other disruption strain with the reference strain (data not shown). This result is consistent with the presence of compensatory mutations, but we cannot exclude the possibility that the different distributions are caused directly by the *dps *deletion, for example by systematically altering the effect of insertions in certain classes of genes. One way to resolve this ambiguity would be to transfer each secondary mutation to a series of re-isolated *dps*-deleted clones. The finding that the effect of the secondary mutations was consistent over a number of such clones would support the interpretation that compensatory mutations did not affect our original results. We are currently in the process of making these constructs and the results will be reported in a future communication. In the meantime, we note that when we omit the *dps*-deleted strain from our analysis of the mean effect of secondary mutations, disruption type still explains a substantial, though marginally non-significant portion of overall variation (*F*_1,9 _= 3.663, *P *= 0.088). Thus, it seems unlikely that compensatory mutations play a major role in driving a lower mean effect of secondary mutations amongst the hub disruption strains.

### Negative relationship between environmental and mutational robustness

It has been proposed that environmental and mutational robustness may be aspects of the same phenomena: resistance of the cell to perturbation [13,18,27,28]. Indeed, the limited experimental evidence available (reviewed in 28) largely supports this view. Therefore, we were surprised to find a negative correlation among disruption strains in their degree of variation in response (i.e. variance robustness) to environmental and mutational stresses, with only one outlying strain (the *fis*-deleted one) disrupting a statistically significant correlation. In view of the relatively small number of disruption strains studied, we are cautious about over-interpreting this relationship. However, in the absence of any conspicuous discrepancies, we think it is worth considering the implications of a negative association between environmental and mutational robustness.

In addition to being robust to short-term perturbations, organisms must be able to adapt to long-term changes in environmental conditions. In this context, the predicted positive correlation between environmental and mutational robustness is considered problematic, because, by limiting the ability of mutations to increase phenotypic variation, robustness will decrease an organism's ability to adapt in the face of future environmental changes (i.e. its evolvability). That is, organisms that are most robust to short-term fluctuations are simultaneously less able to respond to long-term opportunities [10,29-31]. At least one simulation study has demonstrated the action of this trade-off [31]. Our finding of a negative relationship between environmental and mutational robustness suggests that *E. coli *may have found a way around the trade-off, somehow allowing networks to be robust to environmental changes, without insulating them from the effect of the mutational changes that are essential for adaptive evolution. Indeed, in some disruption strains, the average effect of the insertion mutations was positive (though, as noted above, this effect was only significant in the case of the *dps*-deleted strain), despite the same disruptions causing a reduction in environmental robustness. In these cases, the additional mutations cannot be considered as stresses. In our view, this highlights the fact that changes to organism's regulatory networks can provide new evolutionary opportunities. Exploring potential mechanisms underlying these opportunities will be an exciting avenue for future work.

## Conclusion

We tested the expectation that the power-law organization of biological networks confers robustness by comparing the effect of random and hub gene disruptions. We used a simple 'relative measure' approach to separate the topological and biochemical effect of each disruption by comparing the growth rate of disruption strains in stressful and reference environments. We applied stresses externally, by exposing strains to environmental changes, and internally, by introducing additional random mutations. We found that the disruption of random genes had significantly less effect on robustness to environmental perturbation than did disruption of targeted hub genes. However, surprisingly, random disruption strains were less robust to additional mutations than were hub disruption strains. Considered over all disruption strains, the relationship between resistance to environmental and mutational stress was negative, suggesting that different mechanisms of resistance may be involved. These findings support the hypothesis that properties of power-law distributions can play a role in the selection of regulatory network organization.

## Methods

### Mutation strains

The reference strain was a clone of *E. coli *B/r, designated REL606, which has been described previously [32]. Targeted hub disruption strains were constructed by deleting genes encoding global regulators. Genes deleted were: *crp*, *fis*, *dps*, *rpoS *and *relA-spoT*. The first four of these genes directly encode a regulatory gene [20,33-35], *relA *and *spoT *together control the synthesis of the global regulatory molecule (p)ppGpp [36]. Deletions were carried out using previously described suicide vector systems [37,38]. The high number of connections these genes are involved in (table 1) put them in the top 1% of most connected genes in the *E. coli *transcriptional network [16]. We note that the *dps *gene encodes a histone-like protein which mediates stress resistance during starvation through widespread nucleoid reorganization. This mechanism of regulation is not readily amenable to computational analysis but empirical studies have shown that dps does function as a global regulator. Dps affects gene expression [39] and direct comparison of global protein expression between reference and *dps *mutant strains revealed dramatic alteration in the protein profiles with changes in about 10% of detected proteins [40]. Random gene disruption strains were generated using a mini-Tn*10 *transposon derivative having reduced bias in potential insertion sites [41]. Because it was possible that insertions could occur in intergenic regions of the *E. coli *chromosome, we subsequently mapped the position of each insertion mutation. In all cases insertions occurred in coding regions. The gene disrupted by each insertion is (strain designation as in figures 1–3): R1, *tdcB*;R2, *sanA*; R3, *yehB*; R4, *ycfZ*; R5, *ygiZ*; R6, *fecI*; R7, *glxK*. Of these genes only two, *tdcB *and *fecI *are known to play a role as regulators in the *E. coli *transcriptional network: *tdcB *is part of an operon regulated by six transcription factors and *fecI *co-regulates a total of seven genes organized in two operons. We note that the insertion mutations did reduce growth rate relative to the ancestor, indicating they did have some phenotypic effect (see figure 1).

### Environmental stress

Identity and concentration of environmental stresses were chosen on the basis of reducing the growth rate of the ancestral strain by ~50%. The fifteen stresses used and their working concentration are as follows: spectinomycin, 75 μg/ml; erythromycin, 5 μg/ml; D-cycloserine, 12.5 μg/ml; rifampicin, 0.2 μg/ml; chloramphenicol, 0.3 μg/ml; trimethoprim, 0.04 μg/ml; nalidixic acid, 0.3 μg/ml; tetracycline, 0.7 μg/ml; nickel, 1 mM; manganese, 6.25 mM; calcium, 0.156 mM; hydrochloric acid, 0.125% saturated solution; sodium hydroxide, 12.5 mM; ethylenediaminetetraacetic acid (EDTA), 12 mM; sodium chloride, 0.25 M. All stresses were added to their working concentration to Luria-Bertani (LB) medium.

### Generation of strains containing secondary mutations

Thirty strains, each containing one additional mini-Tn*10 *insertion mutation, were made from each of the twelve primary gene disruption strains and from the reference strain. These strains were constructed using a mini-Tn*10 *transposon encoding a different resistance marker than the random disruption strains, allowing direct selection of mutants containing the secondary insertion. The mini-Tn*10 *was transferred into primary disruption strains by conjugation. To ensure that strains containing the secondary mutation were independent, no more than one strain was collected from any one of at least 95 independent conjugation experiments carried out for each primary disruption strain. The possibility of more than one secondary insertion being present in a strain has been shown previously to be very low [25]. To reduce the likelihood of fixing additional mutations during this process, colonies containing the secondary mutation were picked and restreaked as soon as they were visible (approximately 14 hours). Colonies arising following restreaking were grown for 12 hours in liquid LB then stored at -80°C with the addition of glycerol. To ensure this protocol did not bias against slow growing strains we marked colonies visible following the initial colony selection and continued incubation of the selection plate for a further 24 hours. Colonies that arose between these time points were counted and compared to the number present at the earlier time point. Approximately 15 of these slower-growing colonies were restreaked and stored following the same protocol as for the faster growing colonies. No further colonies arose after continued incubation. The final collection of 30 secondary disruption containing strains was chosen to comprise a ratio of strains visible at ~14 hrs and ~14 + 24 hours that accounted for the fraction of colonies visible on selection plates at those time points.

### Estimation of growth parameters

Growth rates of all strains was measured at 37°C in 96 well micro titer plates using a VersaMax (Molecular Devices, Sunnyvale CA) spectrophotometer. Prior to measurement, strains were grown overnight from freezer stocks in 96 well micro titer plates in 100 μl of LB medium, then transferred at a 1:100 dilution to a fresh 96 × 100 μl LB plate and incubated for a further 24 hours. For measurements of environmental robustness, after this second incubation, each strain was used to inoculate two adjacent wells, one a reference well containing only LB, the other containing LB supplemented with one of the fifteen environmental stresses. This plate was incubated in the spectrophotometer and an OD_600 _measurement taken every 5 minutes to allow estimation of growth rate. Each primary disruption strain was present in triplicate in each micro titer plate. Only one stress was applied per plate. Mutational robustness assays were performed similarly, except each primary disruption strain was paired with a derivative of the same strain containing a secondary insertion mutation. In this case, each micro titre plate contained three secondary mutation strains from each primary disruption strain. Three replicate environmental robustness and between two and three mutational robustness assays were performed for each primary disruption strain. Growth rates were calculated using an algorithm that found the steepest ten consecutive measurement points (corresponding to 45 minutes) in natural log transformed OD_600 _measurements. For each disruption strain, environmental or mutational stress was calculated by taking the log ratio of growth rate in the presence of stress over growth rate in the absence of stress. This normalization allows us to estimate the effect of the stress on the primary disruption strain independently of the effect of the disruption itself.

### Statistical methods

Mixed models were run to test the effect of disruption type (hub or random) in the response to environmental and mutational stress. To examine environmental robustness, disruption type and environment were tested individually and as an interaction term, and strain (i.e. disrupted gene) was nested under disruption type. To examine mutational robustness, genotype was nested within disruption type and secondary mutation was nested under strain and disruption type. In all cases, models were fitted using the effect of the stress being examined (relative to effect on the mutation in a reference stress-free environment) on maximum growth rate as the response variable. Disruption type was treated as a fixed factor, genotype, secondary mutation and environment were treated as random factors. To test for the significance of heterogeneity in variance of random factors, a model was fitted with and without a term allowing for differences in variance between the treatments. Specifically, this was achieved by using the "group" option in PROC MIXED to fit different variances to the stress responses of each disruption strain grouped under disruption strain type (i.e. hub or random) (SAS institute Inc., Cary NC) [43]. Likelihood-ratio tests were used to test the significance of the additional factor. The significance of fixed effects were tested using partial F-tests. In the case of the environmental robustness results shown in figure 4, data were normalized to the block average (each block contained all measurements of one environmental stress) for the purposes of presentation.

## Authors' contributions

TFC and APM conceived of and designed the experiments. DS carried out targeted strain constructions, TFC, APM, and AG carried out other strain constructions and performed growth rate experiments. TFC performed statistical analyses. All authors contributed to and approved the final manuscript.

## Supplementary Material

Additional File 1 Supplementary statistical analyses. Detailed analyses of robustness measurements provided in the manuscript. Click here for file

## Figures and Tables

**Table 1:** Effect of hub disruptions on transcriptional regulatory network topology.

Disruption	Connections lost^1^	Average pair-wise network distance^2^
Reference	-	3.965
*crp*	283	4.480
*dps*^3^	2	3.968
*fis*	152	4.040
*relA *&*spoT*	66	4.001
*rpoS*	40	4.017

^1 ^Calculated as the total number of interactions the gene is involved in using the database of regulatory interactions described in Salgado et al (2006) [16]. See also note 3.

^2 ^Calculated as the average pair-wise number of interactions separating all possible pairs of nodes using Pajek network analysis program [42].

^3 ^The Dps protein affects gene expression, mediating changes in about 10% of 300–400 detected proteins [39-40]. Moreover, Dps is involved in massive and non-random reorganization of bacterial nucleoid during stationary phase, leading to global changes in gene expression. The indirect nature of these changes means that analysis of regulatory targets is not readily amenable to conventional computational analysis.

**Table 2:** Summary of significance tests for mean and variance effects of environmental and mutational robustness.

Source^1^	Test statistic^2^	*P*
Environmental robustness:		
Disruption type	3.5	0.064
Disruption type × environment	32.1	<0.001
Strain(disruption type) *variance*	9.6	0.001
Mutational robustness:		
Disruption type	39.4	<0.001
Strain(disruption type) *variance*	0.7	0.201

^1 ^Disruption type was treated as a fixed effect. Environment and strain were treated as random effects.

^2 ^Significance of the fixed effect, disruption type, was tested with a partial F-test. Random effects were tested with likelihood ratio tests.
