# Supplementary material for: Effect of random and hub gene disruptions on environmental and mutational robustness in Escherichia coli
Source: BMC Genomics. 2006 Sep 18;7:237. doi: 10.1186/1471-2164-7-237 (PMC1590030; doi:10.1186/1471-2164-7-237)
Supplement: Additional File 1 — Supplementary statistical analyses. Detailed analyses of robustness measurements provided in the manuscript. [file 1471-2164-7-237-S1.doc]

Supplementary table 1. Anova testing within group variance of random gene disruption strains. Strain is a random effect.

| Source | df | SS | MS | F | *P* |
| --- | --- | --- | --- | --- | --- |
| Strain | 6 | 0.012 | 0.002 | 1.575 | 0.162 |
| Error | 98 | 0.123 | 0.001 | 0.162 |  |
| Total | 104 | 0.135 |  |  |  |

Supplementary table 2. Anova testing within group variance of hub gene disruption strains. Strain is a random effect.

| Source | df | SS | MS | F | *P* |
| --- | --- | --- | --- | --- | --- |
| Strain | 4 | 0.710 | 0.178 | 88.676 | <0.001 |
| Error | 60 | 0.120 | 0.002 | 0.000 |  |
| Total | 64 | 0.831 |  |  |  |

Supplementary table 3. Two-way nested ANOVA influence of disruption type on growth rate over environmental stress. Disruption type is a fixed effect, environment and strain are random effects.

| Source | df | SS | MS | F | P |
| --- | --- | --- | --- | --- | --- |
| Disruption type | 1 | 1.270 | 1.270 | 1.588 | 0.225 |
| Environment | 14 | 93.809 | 6.701 | 21.329 | <0.001 |
| Environment x disruption type | 14 | 4.398 | 0.314 | 3.452 | <0.001 |
| Strain(disruption type) | 10 | 5.785 | 0.579 | 6.357 | <0.001 |
| Error | 497 | 45.231 | 0.091 |  |  |
| Total | 536 | 154.573 |  |  |  |

Supplementary table 4. Anova testing variation between random disruption strains in response to environmental stresses.

| Source | df | SS | MS | F | *P* |
| --- | --- | --- | --- | --- | --- |
| Environment | 14 | 60.743 | 4.339 | 98.050 | <0.001 |
| Strain | 6 | 0.633 | 0.105 | 2.384 | 0.029 |
| Error | 294 | 13.010 | 0.044 | 0.000 |  |
| Total | 314 | 74.386 |  |  |  |

Supplementary table 5. Anova testing variation between hub disruption strains in response to environmental stresses.

| Source | df | SS | MS | F | *P* |
| --- | --- | --- | --- | --- | --- |
| Environment | 14 | 40.806 | 2.915 | 18.363 | <0.001 |
| Strain | 4 | 5.152 | 1.288 | 8.115 | <0.001 |
| Error | 203 | 32.221 | 0.159 | 0.000 |  |
| Total | 221 | 78.838 |  |  |  |

Supplementary table 6. Nested ANOVA testing influence of disruption type on growth rate over mutational stress. Disruption type is a fixed effect, environment and strain are random effects.

| Source | df | SS | MS | F | P |
| --- | --- | --- | --- | --- | --- |
| Disruption type | 1 | 0.123 | 0.123 | 6.022 | 0.034 |
| Strain(disruption type) | 10 | 0.205 | 0.021 | 5.201 | <0.001 |
| Secondary mutation(disruption type, strain) | 348 | 1.398 | 0.004 | 1.283 | 0.007 |
| Error | 432 | 1.352 | 0.003 |  |  |
| Model | 791 | 3.133 |  |  |  |
